# Increase in peg-asparaginase clearance as a predictor for inactivation in patients with acute lymphoblastic leukemia

**DOI:** 10.1038/s41375-024-02153-6

**Published:** 2024-01-29

**Authors:** Merete Dam, Maddalena Centanni, Lena E. Friberg, Daniel Centanni, Mats O. Karlsson, Line Stensig Lynggaard, Inga Maria Johannsdottir, Hilde Skuterud Wik, Johan Malmros, Goda Elizabeta Vaitkeviciene, Laimonas Griskevicius, Helene Hallböök, Ólafur Gísli Jónsson, Ulrik Overgaard, Kjeld Schmiegelow, Stefan Nygaard Hansen, Mats Heyman, Birgitte Klug Albertsen

**Affiliations:** 1 Department of Paediatrics and Adolescent Medicine, Aarhus University Hospital, Aarhus, Denmark; 2 Department of Clinical Medicine, Aarhus University, Aarhus, Denmark; 3 Department of Pharmacy, Uppsala University, Uppsala, Sweden; 4 Department of Paediatric Haematology and Oncology, Oslo University Hospital, Oslo, Norway; 5 Department of Haematology, Oslo University Hospital, Oslo, Norway; 6 Astrid Lindgren Children’s Hospital, Karolinska University Hospital and Department of Women’s and Children’s Health, Karolinska Institutet, Stockholm, Sweden; 7 Vilnius University Children’s Hospital, Vilnius, Lithuania; 8 Vilnius University Hospital, Vilnius, Lithuania; 9 Dept Of Medical Sciences, Haematology, Uppsala University, Uppsala, Sweden; 10 National University Hospital of Iceland, Reykjavik, Iceland; 11 Department of Haematology, Rigshospitalet, University of Copenhagen, Copenhagen, Denmark; 12 Department of Pediatrics and Adolescent Medicine, Rigshospitalet, University of Copenhagen, Copenhagen, Denmark; 13 Institute of Clinical Medicine, Faculty of Medicine, University of Copenhagen, Copenhagen, Denmark; 14 Department of Public Health, Aarhus University, Aarhus, Denmark

**Keywords:** Medical research, Risk factors, Immunology

## Abstract

Asparaginase is an essential component of acute lymphoblastic leukemia (ALL) therapy, yet its associated toxicities often lead to treatment discontinuation, increasing the risk of relapse. Hypersensitivity reactions include clinical allergies, silent inactivation, or allergy-like responses. We hypothesized that even moderate increases in asparaginase clearance are related to later inactivation. We therefore explored mandatory monitoring of asparaginase enzyme activity (AEA) in patients with ALL aged 1–45 years treated according to the ALLTogether pilot protocol in the Nordic and Baltic countries to relate mean AEA to inactivation, to build a pharmacokinetic model to better characterize the pharmacokinetics of peg-asparaginase and assess whether an increased clearance relates to subsequent inactivation. The study analyzed 1631 real-time AEA samples from 253 patients, identifying inactivation in 18.2% of the patients. This inactivation presented as mild allergy (28.3%), severe allergy (50.0%), or silent inactivation (21.7%). A pharmacokinetic transit compartment model was used to describe AEA-time profiles, revealing that 93% of patients with inactivation exhibited prior increased clearance, whereas 86% of patients without hypersensitivity maintained stable clearance throughout asparaginase treatment. These findings enable prediction of inactivation and options for either dose increments or a shift to alternative asparaginase formulations to optimize ALL treatment strategies.

## Introduction

Acute lymphoblastic leukemia (ALL) is the most common childhood cancer. Asparaginase is a cornerstone in the treatment of ALL contributing to the high survival rate [1]. Debates persist concerning the most effective treatment regimen in terms of the quantity of doses, dosage, and timing of asparaginase extending to the broader treatment strategy [2]. Within the ALLTogether treatment protocol, the dosing and number of doses vary based on age and risk groups (EudraCT no.2018-001795-38). *Escherichia coli L-asparaginase* can be covalently linked to polyethylene glycol (peg-asparaginase) in order to decrease immunogenicity of the enzyme and to prolong its half-life [3]. However, the most common cause of truncation of peg-asparaginase treatment is still hypersensitivity (8–15%) [1, 4–6], and truncation of asparaginase treatment has been shown to increase the risk of relapse [1, 7–10]. Asparaginase hypersensitivity is defined as; clinical allergy ranging from a mild rash to anaphylaxis and silent inactivation (SI) with absence of clinical symptoms. Both conditions are associated with inactivation of asparaginase enzyme activity (AEA) and switching treatment to another asparaginase formulation is indicated [7, 11, 12] Additional allergy-like reactions is a type of asparaginase hypersensitivity with more varying symptoms mimicking true allergies, but - importantly - the AEA is unaffected and peg-asparaginase treatment may proceed if the symptoms are not too pronounced [3, 13].

Real-time therapeutic drug monitoring (TDM) of AEA is an important tool to ensure optimal treatment and to identify patients with inactivation of the drug providing a possibility to switch asparaginase preparation[3, 4, 14]. Additionally, TDM makes it possible to distinguish true allergies with inactivation from allergy-like reactions[3, 4, 6, 13, 15].

AEA trough concentration (C_trough_) ≥ 100 IU/L two weeks after an administration of peg-asparaginase has been defined as the therapeutic activity target level to ensure complete asparagine depletion[6, 11, 13, 16]. Previous studies showed that the number of peg-asparaginase doses could be reduced while maintaining high survival rates and reducing toxicity significantly[8, 17–19]^,^ resulting in less asparaginase treatment in many contemporary protocols compared with previous protocols. Consequently, it is crucial that all doses of peg-asparaginase are effective.

The inactivation of asparaginase has been a long-standing subject of interest, and numerous explanations have been proposed [20]. It has been hypothesized that asparaginase inactivation follows a classical immune response with initial IgM formation followed by affinity maturation and isotype switching to the IgG and IgE subclasses [21, 22] which could indicate that early changes in clearance could be detected. The detection of inactivation-based changes in clearance using AEA is obscured by drug accumulation, intra-patient variability and increased intra-dose clearance due to depegylation. Hence, the application of pharmacokinetic analysis becomes necessary to delineate these elements [23]. Wurthwein et al. [24] characterized the standard elimination in peg-asparaginase AEA-time profiles, however, the study design only included two consecutive doses and samples indication inactivation were excluded. Changes in clearance over the treatment period due to an inactivation response was not part of the scope of the study.

Prediction of inactivation from AEA levels at an early stage would provide an opportunity to decrease the risk of potentially life-threatening allergic reactions and to switch to another asparaginase preparation at the optimal time to ensure effective treatment. Furthermore, prediction of AEA-levels might be useful to decrease the peg-asparaginase dose in patients with expected high AEA, which may be associated with an increased risk of certain toxicities (pancreatitis and avascular necrosis) [25].

The overall aim of this study was to assess whether AEA can be used to identify patients with future peg-asparaginase inactivation. To achieve this goal, peg-asparaginase enzyme activity was monitored in patients included in the ALLTogether pilot protocol, the relationship between measured AEA were related to timing of the dose and the probability of an inactivation event and a pharmacokinetic analysis was conducted to characterize the trend and variability in AEA-time profiles and to verify whether a change or an increase in clearance are associated with the development of an inactivation response.

## Methods

### Study population

The included patients were aged 1–45 years and diagnosed with de novo Philadelphia chromosome negative B-cell precursor or T-cell ALL. Patients were treated according to the ALLTogether pilot protocol conducted in Denmark, Iceland, Lithuania, Norway and Sweden from November 2018 to February 2022. Patient and disease characteristics, treatment and toxicity information were retrieved from the ALLTogether study database (Castor) and by contact with the treating centers.

### Risk-stratification and treatment

In the ALLTogether pilot protocol, patients were stratified into four main risk groups; standard-risk (SR), intermediate-risk low/high (IRL/IRH), or high-risk (HR) based on clinical features; age, immunophenotype (B-cell and T-cell), cytogenetics and treatment response, by evaluation of measurable residual disease at day 29 and day 71/78 (Supplementary 1).

Peg-asparaginase was used as the standard preparation. The dose was adjusted according to age (1500 IE/m^2^ < 16 years and 1000 IE/m^2^ ≥ 16 years) and was administered intravenously (IV) or intramuscularly (IM, mostly adults) over one hour. Administration of peg-asparaginase started from treatment day 4 during the induction phase for all patients. However, following amendment of September 9^th^, 2020, ALLTogether pilot protocol version 2.2, patients aged 25 years and older (*n* = 2) received their first dose of peg-asparaginase on day 18 of the treatment protocol. Peg-asparaginase was administered with two-week intervals to maintain continuous depletion of asparagine for a duration of 8–20 weeks which varied based on the prescribed number of doses according to risk group (SR 4 doses, IRL 5 doses, IRH 8 doses and HR a maximum of 10 doses) (Supplementary Table S2, Supplementary 1). Premedication was not recommended.

The ALLTogether pilot protocol was approved by the National Medicines Agencies (EudraCT no.2018-001795-38) and national or regional ethics committees in each participating country. The study was registered at ClinicalTrials.gov (NCT03911128). All patients and/or caregivers gave consent according to the Declaration of Helsinki.

### AEA sampling and TDM

Sampling for AEA measurements on the ALLTogether pilot protocol was planned as follows; at baseline, on day 1, 4, 7, 11 and 14 after the first dose of peg-asparaginase and 14 days after the subsequent doses. C_trough_ was defined as the AEA level 14 ± 2 days after a peg-asparaginase administration [6, 16, 26]. Lower level of detection (LLOD) was 0.5 IU/L and inter-day variation 2.5-6%. In case of >1 sample in the defined timeslot (day 12–16 after previous dose) the earliest sample in the timeslot was selected for statistical analyses. All samples were included in the pharmacokinetic analyses.

Analysis of AEA, using a validated L-aspartic *β*-hydroxamate assay (AHA) [27], was centralized for all countries in the Nordic Society of Paediatric Haematology and Oncology (NOPHO) at the Asparaginase laboratory at Aarhus University Hospital. AEA levels were measured in real-time and recommendations about continuation, switch or additional sampling were provided to the responsible clinicians through an online database (REDCap electronic data capture tools hosted at Aarhus University) [28, 29]. In REDCap all patients were registered with a unique record-ID with an event for all AEA samples. The clinicians provided information about peg-asparaginase administration date and potential toxicities regarding a specific dose and sample. Missing information was retrieved by direct contact with the treatment sites. The total turnaround time was between 4 and 7 days.

### Inactivation

Clinical allergy was graded as mild or severe according to the Consensus Definitions by the Ponte Di Legno Working Group [4, 11]. Silent inactivation was defined as C_trough_ AEA < LLQ 14 ± 2 days after a peg-asparaginase administration. Allergy-like reactions were defined as varying symptoms of intolerance e.g., vomiting, abdominal pain and/or rash but with C_trough_ > 100 IU/L if infusion was completed [11]. In case of an allergic reaction where the infusion was stopped, the amount of administered peg-asparaginase was registered and extra sampling immediately after interruption of the infusion and within a few days were recommended to verify inactivation. If over half the dose was given, an extra day-seven sample was recommended after an allergic reaction. If AEA on day seven exceeded 100 IU/L, another peg-asparaginase dose would be proposed. In case of inactivation, the recommendations were to replace the remaining doses with native *Erwinia* chrysanthemi-derived asparaginase (20,000 IU/m^2^/dose) administered every second day, which means that one dose of peg-asparaginase was substituted with seven doses of *Erwinia*-derived asparaginase.

### Statistical analyses

A linear mixed model was used to evaluate the relationship between mean AEA C_trough_ levels and dose number with dose number entered as a categorical variable. A random effect (intercept) per patient was included to model the dependency between measurements from the same patient. Results were reported by no inactivation versus inactivation and stratified by route of administration (IM and IV administration), age (</≥16 years) and separated for time of allergic reaction.

A logistic regression model was used to model the relationship between AEA levels at day seven and inactivation after the first peg-asparaginase dose with AEA levels entered via a restricted cubic spline with four knots. This enabled us to evaluate the predictive performance of AEA levels at day seven on the risk of asparaginase inactivation.

### Pharmacokinetic model

A population pharmacokinetic model was developed based on all the available AEA measurements, using the nonlinear mixed effects software NONMEM version 7.4.4 (ICON Development Solutions) [24]. The pharmacokinetics of peg-asparaginase are characterized by an increased clearance within the dosing interval as a consequence of depegylation, in addition to an increased clearance over the treatment period in some subjects due to development of inactivation. To capture these patterns, several structural models were assessed to describe the quantified data (Supplementary data 2. Structured models) [24], simultaneously accounting for readings below the lower level of quantification (LLQ) (≤5 IU/L) [30]. The model estimated the probability that patients belonged to one of two subpopulations; i.e. I) constant clearance or II) increased clearance over time. In a posthoc step, each patient was assigned to the group with their highest probability. The sensitivity and specificity of the model to identify increased clearance in patients that developed an inactivation was assessed. Inter-individual variability was evaluated on all structural parameters. To account for different body sizes, pharmacokinetic parameters were adjusted to body weight using allometric scaling [31]. The impact of age was additionally assessed on the clearance parameters.

## Results

### Patient characteristics

**Fig. 1:**
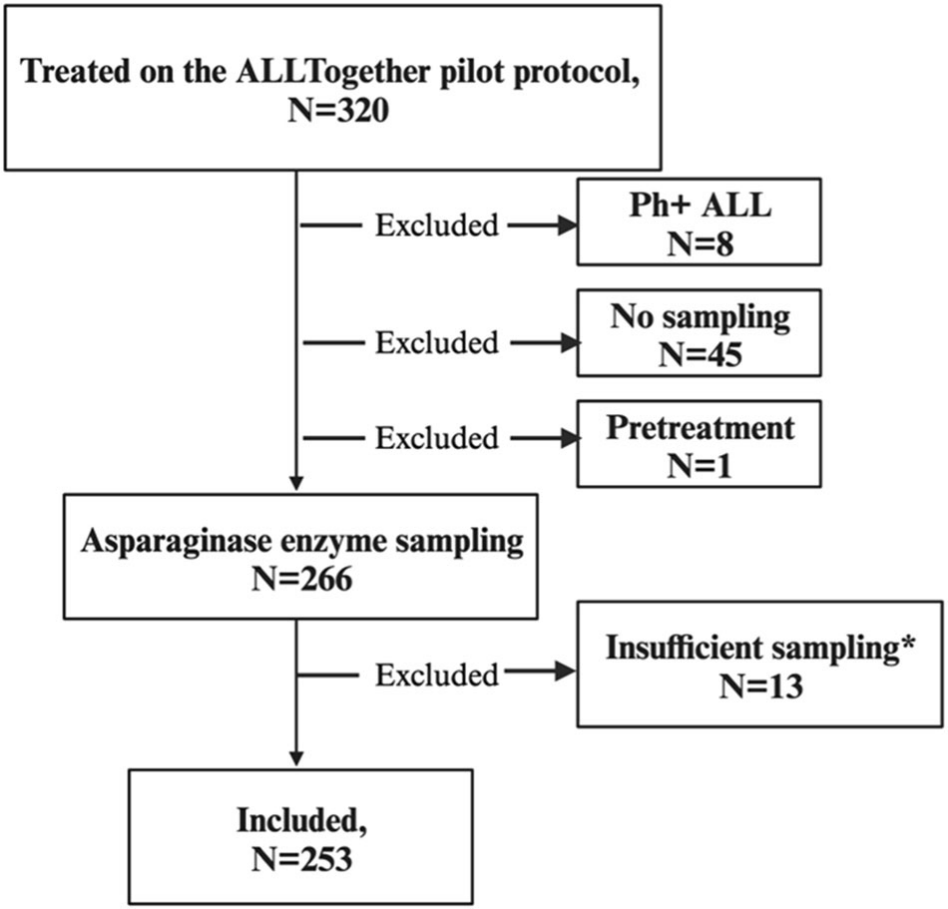
Flowchart illustrating inclusion and exclusion. Ph+ = Philadelphia chromosome positive acute lymphoblastic leukemia. * Patients with one sample or only samples outside the defined timeslot 14 +/− 2 days from previous dose. One patient had plasmapheresis and samples were not reliable.

A total of 320 patients were included in the ALLTogether pilot protocol during the study period. Following the exclusion of patients with outlier AEA (>16 days after previous dose) and/or insufficient sampling ($\le$1 sample), 253 patients (79%) with 1631 AEA measurements (mean 6.5 samples per patient) were included in the statistical analyses, out of these, 86.6% (*n* = 219) were children 1–17 years and 13.4% (*n* = 34) young adults 18–45 years (Fig. 1).

The median age at diagnosis was 5.9 years and 61.3% of the patients were male. Patients were stratified to the following risk groups; SR 35.6%, IRL 24.9%, IRH 31.6% and HR 7.1%. The majority of patients (229/253, 90.5%) were treated with IV peg-asparaginase. IM administration was used in 3/219 children (1.4%) and 22/34 (61.1%) adults. Baseline characteristics of the patients are described in Table 1. Dosing of peg-asparaginase was 1500 IE/m^2^ for 80.5% of patients (<16 years) and 1000 IE/m^2^ for 19.5% of patients (≥16 years).

**Table 1:** Baseline characteristics of patients.

	Cohort, *n* (%)	Inactivation, *n* (%)	No inactivation, *n* (%)
Patients	253	46 (18.2)	207 (81.8)
Sex			
Female	98 (38.7)	13 (28.3)	86 (41.5)
Male	155 (61.3)	33 (71.7)	122 (58.5)
Age, median, years	5.88	6.32	5.86
1–9.9	165 (65.2)	31 (67.4)	134 (64.7)
10–17.9	54 (21.3)	12 (26)	42 (20.3)
18–45	34 (13.4)	3 (6.5)	31 (15.0)
Risk group			
SR	90 (35.6)	17 (37)	73 (35.3)
IR-Low	63 (24.9)	11 (23.9)	52 (25.1)
IR-High	80 (31.6)	15 (32.6)	65 (31.4)
HR	19 (7.5)	3 (6.5)	16 (7.7)
NA^a^	1		1
Immunophenotype			
B-cell ALL	227 (89.7)	43 (93.5)	184 (88.9)
T-cell ALL	24 (9.5)	2 (4.3)	22 (10.6)
Mixed	2 (0.8)	1 (2.2)	1 (0.5)

^a^One patient died before risk stratification.

Sampling days 1–16 after the first dose and AEA C_trough_ (12–16 days) after doses 2–7 were included in the basic statistical evaluation. Samples from day 7 after the first dose were used in the logistic regression model. AEA C_trough_ after dose 8 were too few to be included.

### Asparaginase enzyme activity and inactivation

Of 253 patients, 46 (18.2%) experienced hypersensitivity with inactivation during peg-asparaginase treatment; 36/46 patients (78.3%) developed clinical allergy, of those 23/36 patients (63.9%) had severe allergy and 13/36 patients (36.1%) had mild reactions. For all patients with IV administration and allergic symptoms, the reactions were observed during the infusion. None were fatal. Silent inactivation was found in 10/46 (21.7%). One patient had an allergy-like reaction. Two patients who experienced allergic reactions were excluded from the linear mixed model because samples were not collected within the designated timeframe following any dose. Both patients remained in the pharmacokinetic model with AEA measurements still within 14 days but outside the desired range of 14 +/− 2 days (C_trough_).

Clinical allergy occurred during administration of the third (*n* = 11/36, 30.6%), the fourth (*n* = 19/36, 52.8%) or the fifth (*n* = 6/36, 16.7%) dose of peg-asparaginase. For 23 of 36 (63.9%) of the patients with clinical allergy, AEA C_trough_ were available reflecting the AEA level after the previous dose given. In all cases C_trough_ was <LLQ. For the remaining 13 patients (36.1%) with clinical allergy no AEA C_trough_ before the allergy-triggering dose was available. However, in three of these cases, C_trough_ at an earlier stage during treatment showed AEA C_trough_ < LLQ. SI was seen as C_trough_ < LLQ after the second dose in two cases (20%), after the third dose in six cases (60%) and after the fourth dose in two cases (20%).

### Statistical analyses

**Fig. 2:**
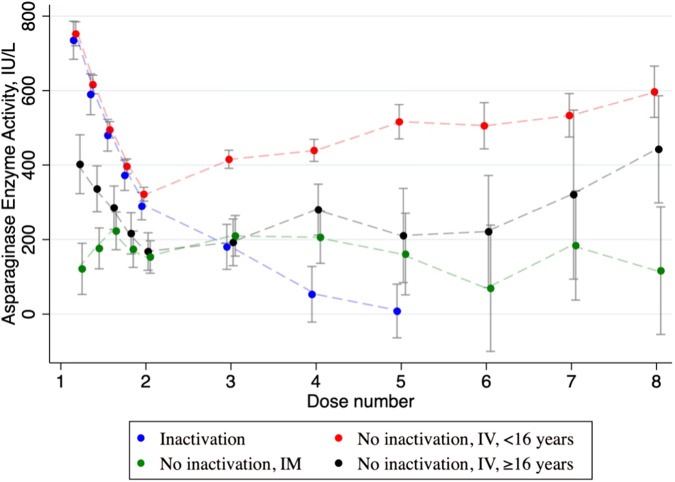
AEA measurements and 95% CI over time in the groups; IM administration, IV administration, </≥16 years and inactivation. AEA measurements day 1,4,7 and 11 after first dose and AEA C_trough_ after every dose of peg-asparaginase. Patients with inactivation are illustrated as the blue line (including one patient with IM administered PEG-asparaginase and three patients ≥16 years) and patients in the non-inactivation group as the red, black and green line according to route of administration and age group.

The AEA C_trough_ for patients with and without inactivation are illustrated in Table 2 and Fig. 2. The inactivation group consisted of 45 patients with AEA C_trough_ in the defined time slot (12–16 days after previous dose); 42 patients were under 16 years treated with IV peg-asparaginase (93.3%), 2 patients ≥16 years treated with IV peg-asparaginase (4.4%) and one patient (<16 years) treated with IM peg-asparaginase (2.2%).

**Table 2:** Mean AEA C_trough_ after the first four doses of PEG-asparaginase in the different groups.

	AEA C_trough_ IU/L						
	No inactivation			Inactivation			
	IV		IM	IM and IV	IV		IM
Dose	AEA (samples, *n*) Patients = 184		AEA (samples, *n*) Patients = 24	AEA (samples, *n*) Patients = 45	AEA (samples, *n*) Patients = 42	AEA (samples, *n*) Patients = 2	AEA (samples, *n*) Patients = 1
	<16 years	≥16 years	1 < 16 years ≥23		<16 years	≥16 years	<16 years
1	322 IU/L (156)	168 IU/L (18)	142 IU/L (22)	295 IU/L (43)	301 IU/L (40)	70 IU/L (2)	487 IU/L (1)
2	413 IU/L (142)	189 IU/L (13)	199 IU/L (17)	193 IU/L (38)	203 IU/L (36)	0 IU/L (1)	0 IU/L (1)
3	440 IU/L (130)	270 IU/L (15)	210 IU/L (11)	94 IU/L (26)	94 IU/L (26)	–	
4	523 IU/L (73)	197 IU/L (6)	164 IU/L (8)	0 IU/L (10)	0 IU/L (9)	0 IU/L (1)	

Inactivation group: 44 patients were treated with IV PEG-asparaginase; 42 patients were <16 years (1500 IE/m^2^/dose), and two patients were ≥16 years (1000 IE/m^2^/dose) and one patient had PEG-asparaginase IM (age <16 years).

No inactivation group: 184 patients were treated with IV PEG-asparaginase; 165 patients were <16 years (1500 IE/m^2^/dose), and 19 patients were ≥16 years (1000 IE/m^2^/dose). 24 patients were treated with IM PEG-asparaginase; 1 patient was <16 years (1500 IE/m^2^/dose) and 23 patients were ≥16 years (1000 IE/m^2^/dose).

Mean AEA C_trough_ after the first IV peg-asparaginase administration was 322 IU/L (95% Confidence Interval (CI): 303–340 IU/L) <16 years (all were IV treated), 168 IU/L ≥ 16 years in the non-inactivation group (all were IV treated) and 295 IU/L in the inactivation group (97.8% IV treated, 2.2% IM treated) (Table 2).

Mean AEA C_trough_ after the second IV administration was 203 IU/L in patients <16 years with inactivation. In patients <16 years without inactivation, the mean AEA C_trough_ was 413 IU/L and 189 IU/L in patients ≥16 years. In patients <16 years mean AEA C_trough_ after the 2^nd^ dose of peg-asparaginase (AEA 203 IU/L) was significantly lower for patients with inactivation than without inactivation (AEA 413 IU/L) (*p* < 0.001) (Table 2). Meaningful statistical analyses were not feasible due to the limited number of patients and samples in the group of individuals aged ≥16 years.

**Fig. 3:**
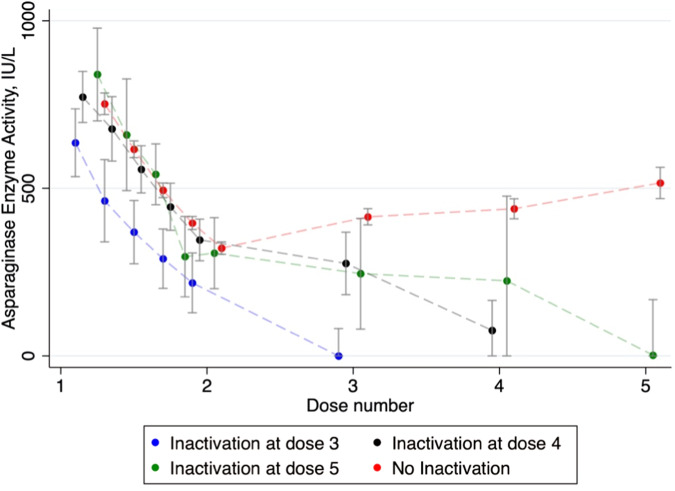
AEA measurements and 95% CI over time in the groups; Allergic reaction on 3^rd^ dose, 4^th^ dose, and 5^th^ dose compared to no inactivation. AEA measurements day 1,4,7 and 11 after first dose and AEA C_trough_ after every dose of peg-asparaginase for patients <16 years (IV administration). Patients without inactivation are shown in red, those experiencing allergic reactions on the 3^rd^ dose in blue, on the 4^th^ dose in green, and on the 5^th^ dose in black.

The overall mean AEA C_trough_ across the first four doses in the non-inactivation group of IV patients was in range 322–516 IU/L for patients <16 years, and 168–211 IU/L in patients ≥16 years. Finally, mean AEA C_trough_ was 179 IU/L in the group of patients treated with IM peg-asparaginase (mean age 27.1, (3–44.8)) (Fig. 2). Considering the timing of allergic reaction (dose 3, 4, or 5), the variation in AEA C_trough_ following the second dose continues to be distinct across all three time points for the allergy (Fig. 3).

**Fig. 4:**
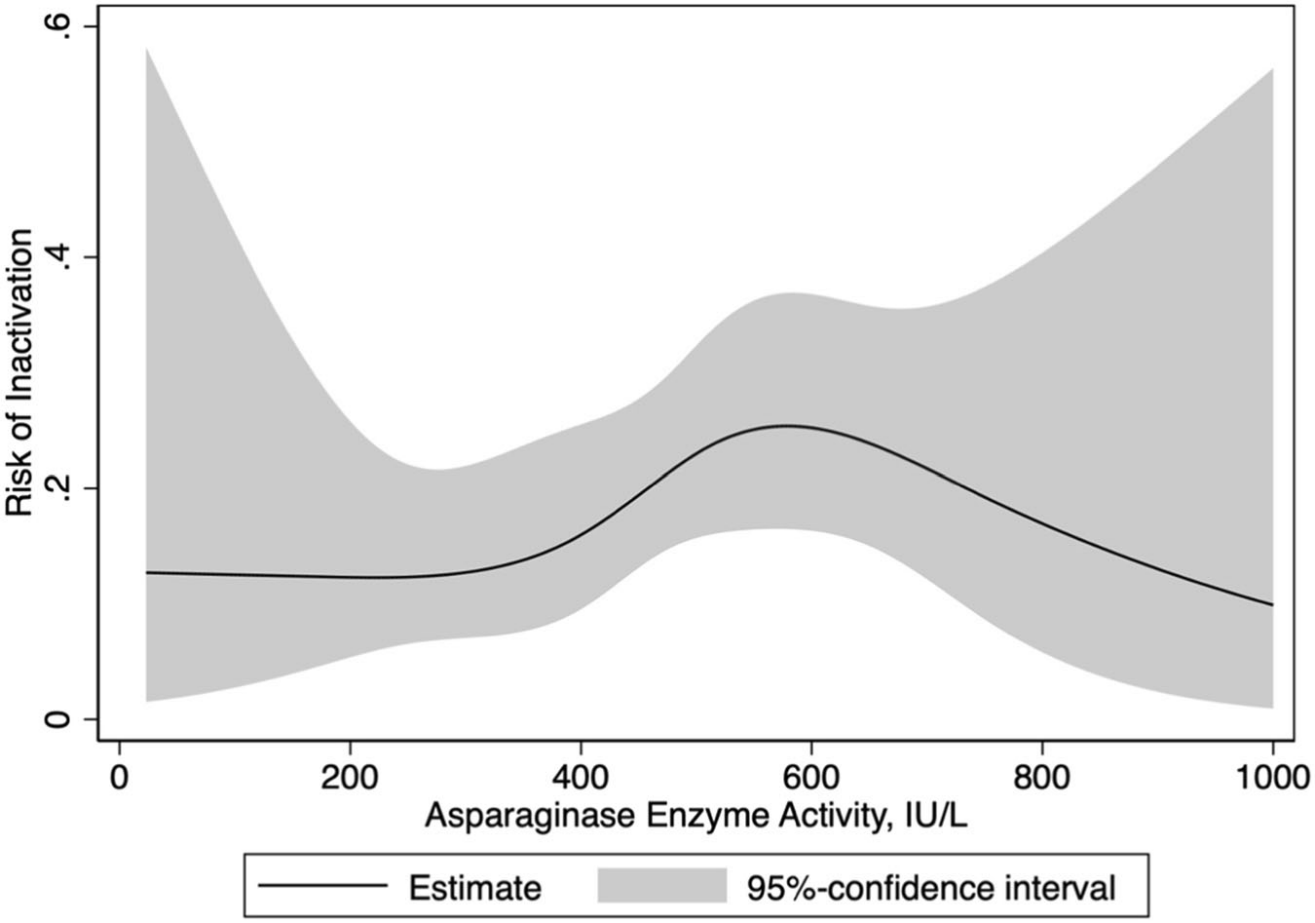
Risk of inactivation of peg-asparaginase. Logistic regression model with AEA seven days after first dose.

The logistic regression analysis (Fig. 4) examined the relationship between the level of AEA measured seven days after the initial dose and the likelihood of inactivation. When evaluating an AEA level of <200 IU/L seven days after the first dose of peg-asparaginase, the risk of inactivation was estimated to be 15% (95% CI: 5.4–25.8). The analysis did not find a statistically significant association between AEA levels at that specific time point and the risk of inactivation.

### Pharmacokinetic model

A pharmacokinetic transit model with a chain of 10-compartments captured the AEA data the best (Supplementary 2). A first-order absorption rate constant was added to describe the IM data. Initial clearance (CL_initial_) for each compartment represented peg-asparaginase clearance at baseline, whereas clearance induced (CL_induced_) out of the last compartment explained the increased clearance between and over dosing occasions. Volume of distribution was estimated at 4.81 L/70 kg (i.e., 1.71 L/25 kg, 3.44 L/50 kg) and CL_initial_ at 0.20 L/day/70 kg (i.e., 0.09 L/day/25 kg, 0.16 L/day/50 kg).

The pharmacokinetic model classified 28% of patients to have increased clearance over time, whereas 72% of patients were classified to have stable clearance over the dosing period. Most patients that developed an inactivation over the course of treatment were classified as having increased clearance over time (93% increased clearance, 7% stable clearance), while most patients without inactivation were classified as having constant clearance over the dosing occasions (86% stable clearance, 14% increased clearance). Based on these results, pharmacokinetic model classifies patients with inactivation based on increased clearance with a sensitivity of 93% and a specificity of 86%.

## Discussion

The aim of this study was to investigate the comprehensive pharmacokinetics of peg-asparaginase and to assess the significance of AEA measurements in predicting inactivation and potentially life-threatening allergic reactions among patients diagnosed with ALL who were undergoing treatment with peg-asparaginase. AEA levels were measured in a specific patient cohort comprising individuals aged 1–45 years with ALL, who received peg-asparaginase treatment in accordance with the ALLTogether pilot protocol implemented in the Nordic and Baltic regions. We demonstrated that a pharmacokinetic model based on the AEA could estimate the probability that patients belonged to one of two subpopulations of either increased or constant clearance over the treatment period. Patients allocated to the increased clearance group over dosing occasions were frequently individuals who experienced inactivation, while those assigned to stable clearance were less prone to inactivate. The difference in clearance was present from the period between 2^nd^ and 3^rd^ dose administered in patients inactivating at 3^rd^ as well as 4^th^ and 5^th^ dose (Fig. 3).

This study presents a pioneering methodology for predicting inactivation by employing a pharmacokinetic model to identify early alterations in clearance. Notably, our investigation is grounded on a robust dataset comprising a substantial number of samples. The adherence to TDM of asparaginase within the NOPHO consortium was exceptional, resulting in near-complete sampling and empowering both statistical and pharmacokinetic analyses. This remarkable compliance is the primary strength of our study. Moreover, the availability of high-quality data pertaining to clinical symptoms further reinforces the study’s findings.

In studies including TDM no difference has been found in the incidence of inactivation when AEA measurements have been used to compare IM and IV administration of peg-asparaginase.^(4, 32)^ Overall, the incidence of inactivation (18.2%) in this study was high compared with concurrent protocols [4, 32] This might be explained by extensive sampling and a high compliance rate or by some type of change in the peg-asparaginase formulation, but this must be considered as speculative. The number of patients (*n* = 45) excluded due to missing ethical approval for sampling in their country did not participate with sampling for TDM measurements making the assessment of the incidence somewhat uncertain. The majority of such patients (56%) were adults, who are generally considered to have a lower incidence of inactivation of asparaginase [33]. Consistent with literature only three of the 46 patients with inactivation in this study were ≥16 years (6.5%).

The mean AEA C_trough_ in the group of patients without inactivation treated with IV peg-asparaginase <16 years was high (322–516 IU/L over the first four doses). In the DCOG and Dana Farber 00–01 studies, attempts were made to reduce or increase the dosage of asparaginase based on enzyme activities. These studies demonstrated unchanged survival rates with the potential for dose reduction [6, 34]. Thus, the higher C_trough_ values for patients <16 years in this cohort indicate that dose reductions may be possible without compromising the efficacy of the asparaginase treatment assuming that higher C_trough_ is not correlated to a better outcome. Higher AEA-values might also be associated with an increased risk of certain toxicities [3, 6, 17].

Previous studies have shown that some patients developed inactivation after the first dose of peg-asparaginase leading to the hypothesis that anti-peg antibodies were present before the start of treatment [5, 35–38]. In the present study all patients had AEA C_trough_ > 100 IU/L after the first dose, which suggests that sensitization to peg-asparaginase occurred after the first administration. Additionally, sampling between dose 1 and 2 showed no significant difference in mean AEA C_trough_ (Figs. 2 and 3) in the groups, supporting that most inactivation were seen following second dose or later. The variation in the triggering dose in different treatment protocols could potentially be attributed to the simultaneous administration of dexamethasone during induction, which may delay the immune response and the occurrence of inactivation reactions [39] and/or the early introduction of peg-asparaginase treatment compared with introduction of peg-asparaginase post-induction e.g. the NOPHO ALL2008 protocol [40] and the CoALL 08–09 [41].

The remarkable sensitivity (93%) and specificity (86%) observed in the pharmacokinetic model highlight its potential value for clinicians. By leveraging this model, healthcare professionals can gain valuable insights to proactively prepare for potential adverse reactions. This preparation may involve extending the infusion duration, implementing premedication, or exploring desensitization techniques. While completely averting inactivation remains a challenge, delaying the reaction opens the door to administering more effective peg-asparaginase doses before a switch. This intervention also offers the potential to act proactively before a life-threatening allergic reaction occurs, thereby providing a means to mitigate distressing experiences for both patients and their families. Importantly, these measures may contribute to optimizing peg-asparaginase treatment and ensuring favorable survival rates.

Few patients (*n* = 18, 7%) demonstrated induced clearance in the pharmacokinetic model but did not develop an allergic reaction during the peg-asparaginase treatment. This could be explained by the low number of peg-asparaginase doses administered according to the risk group (SR patients = 4 doses), which might lead to completion of peg-asparaginase treatment before the full allergic reaction occurred.

Additionally, it is essential to assess the quantity and precise timepoints of pharmacokinetic samples needed to anticipate inactivation events. In our study, the timing of extended sampling was determined by existing evidence. The time point (seven days after first dose) in the logistic regression model showed no value in predicting inactivation why extended sampling for AEA should occur shortly and less than 14 days after administration of the second dose. We strongly recommend incorporating this approach in future studies. Additionally, the implementation of model-based optimal design can offer valuable support in determining the appropriate sampling times [42]. Increasing the number of samples (day four and/or seven) following the second dose is likely to enhance the sensitivity for detecting accelerated clearance as an early indication of impending inactivation, allowing for timely adjustments to the preceding dose or appropriate adjustments to the upcoming dose. Moreover, an additional factor that impacts the timing of hypersensitivity reactions is the presence of breaks or extended intervals between doses [4]. This is supported by unpublished data from the current ALLTogether1 main protocol. A pause in the peg-asparaginase treatment during consolidation 1 has been introduced due to an unacceptable frequency of acute toxicities (unpublished data). Despite the pause in the peg-asparaginase treatment between the second and third doses for patients <25 years, the model is anticipated to detect increased clearance shortly after the second dose. Lastly, although outside of the scope of this project, the pharmacokinetic model could be useful to enable dose reductions in patients that are far above the treatment threshold, potentially resulting in reduced treatment costs and most importantly perhaps reducing the incidence of asparaginase-related toxicities. However, more studies are needed to support this.

One limitation of this study, as well as in other relevant literature, is the potential risk of misinterpreting inactivation reactions. In cases where a severe allergic reaction occurred during infusion, the administration was often terminated prematurely, resulting in the patient receiving only a fraction of the intended dose. This situation makes it challenging to accurately assess the subsequent AEA C_trough_ when it falls below LLQ. Within this study, 26 out of 36 patients who experienced an allergic reaction had AEA C_trough_ values below the LLQ prior to the reaction. Additionally, the majority of these reactions were severe, providing reasonable grounds to assume the presence of true inactivation [13]. However, in 10 out of 36 patients, AEA C_trough_ data were not available to reflect the previously administered dose, potentially leading to misdiagnosis if only a minimal amount of the dose was given. Nonetheless, the severity (seven severe, three mild) and timing of the allergic reactions argue against the likelihood of allergy-like reactions [3, 13]. Future studies are likely to place greater emphasis on maintaining patients on peg-asparaginase, which could potentially influence the treatment strategy in situations of uncertainty, favouring the administration of an additional dose of peg-asparaginase with close TDM supervision. This approach could also involve considering pre-medication and adjusting the infusion duration.

In general, the patient groups ≥16 years, as well as those who received IM peg-asparaginase treatment, were limited in size. This limited sample size within these subgroups led to reduced statistical power and made it impossible to compare the level of AEA between those who experienced inactivation and those who maintained sufficient activity.

## Conclusion

In conclusion, this study utilized a pharmacokinetic model based on AEA measurements to predict inactivation in patients undergoing peg-asparaginase treatment for ALL. The model effectively distinguished patients with increased clearance, who were more likely to experience inactivation, from those with stable clearance. This distinction was evident as early as immediately after 2^nd^ dose in patients who later experienced inactivation.

This methodology represents a pioneering approach for predicting inactivation by identifying early changes in clearance. It offers clinicians a valuable tool to proactively manage treatment, switch asparaginase formulation timely, and potentially prevent ALL relapses.

### Supplementary information

Supplementary 1

Supplementary 2

## Data Availability

The datasets generated during and/or analyzed during the current study are available from the corresponding author on reasonable request.
