# Supplementary material for: Increase in peg-asparaginase clearance as a predictor for inactivation in patients with acute lymphoblastic leukemia
Source: Leukemia. 2024 Jan 29;38(4):712–9. doi: 10.1038/s41375-024-02153-6 (PMC10997509; doi:10.1038/s41375-024-02153-6)
Supplement: Supplementary file 1 — Supplementary 1 [file 41375_2024_2153_MOESM1_ESM.docx]

| Table S1: Centers Participating in the ALLTogether Pilot Study | | | | |
| --- | --- | --- | --- | --- |
| Country | Center | Principal Investigators | Patient Type | Patients Included, N= 266 |
| Denmark | Aarhus University Hospital | Birgitte Klug Albertsen^a^, Merete Dam / Ingolf Mølle | Children/Adults | 11/7 |
| Denmark | Rigshospitalet | Bodil T. Als-Nielsen /Ulrik Malthe Overgaard^a^ | Children/Adults | 32/5 |
| Denmark | Aalborg University Hospital | Steen Rosthøj | Children | 6 |
| Denmark | Odense | Peder Skov Wehner | Children | 11 |
| Sweden | Göteborg | Jonas Abrahmsson / Hege Garelius | Children/Adults | 15/6 |
| Sweden | Stockholm | Johan Malmros^a^ / Joel Joelsson | Children/Adults | 25/1 |
| Sweden | Linköping | Hartmut Vogt | Children | 8 |
| Sweden | Uppsala | Arja Harila-Saari / Helene Hallböök^a^ | Children | 6/0 |
| Sweden | Lund | Anders Castor | Children | 20 |
| Sweden | Umeå | Ulrika Norén Nyström / Antonio Izarra | Children/Adults | 9/1 |
| Norway | Bergen | Anita Andrejeva/ Waleed Majeed Mohammed | Children/Adults | 10/1 |
| Norway | Oslo Universitetssykehus, Rikshospitalet | Inga Maria Johannsdottir^a,^ /Hilde Skuterud Wik^a^ | Children/Adults | 19/7 |
| Norway | Stavanger | Waleed Majeed Mohammed | Adults | 1 |
| Norway | Trondheim/St. Olav | Bendik Lund / Petter Quist Paulsen | Children/Adults | 5/2 |
| Lithuania | Vilnius | Goda Vaitkeviciene^a^ / Laimonas Griskevicius^a^ | Children/Adults | 42/11 |
| Iceland | Reykavik | Ólafur Gísli Jónsson^a^ | Children | 5 |
| ^a^National Principal Investigators | | | | |

# **Supplementary 1 Materials**

**Other study Participants:**

Maddalena Centanni, Daniel Centanni, Lena E. Friberg, Mats O. Karlsson (all from Uppsala, Sweden).

Stefan Nygaard Hansen (Aarhus, Denmark)

Line Stensig Lynggaard (Aarhus Denmark)

Kjeld Schmiegelow (Copenhagen, Denmark)

Mats Heyman (Stockholm, Sweden. Chief Investigator of the ALLTogether Trial)

| **Table S2: Treatment Details in ALLTogether Pilot Protocols for Patients Stratified by Risk Group** | | | | | |
| --- | --- | --- | --- | --- | --- |
| **ALLTogether Pilot Protocol, standard risk** | | | | | |
| **Treatment phase**  Treatment week | **Induction**  Week 0–4 | **Consolidation I**  Week 5–10 | **Consolidation II**  Week 11–17 | **Delayed intensification**  Week 18–24 | **Maintenance** |
| **Peg-asparaginase treatment^a^**  Doses planned  Treatment week no. | 2 doses  1 + 3 | 2 doses  5 + 7 |  |  |  |
| **Other treatment** | INDUCTION A^b^: Dexamethasone (po)  Vincristine (iv)  Methotrexate^c^ (Intrathecal)  INDUCTION B^c^:  + Daunorubicine (iv) | 6-Mercaptopurine (po)  Cytarabine (iv)  Cyclophosphamide (iv)  Methotrexate^c^ (Intrathecal)  Vincristine (iv) | 6-Mercaptopurine (po)  Methotrexate (high dose, iv)  Methotrexate^c^ (Intrathecal) | Dexamethasone (po)  Vincristine (iv)  Methotrexate^c^ (Intrathecal)  6-Mercaptopurine (po)  Cytarabine (iv) | 6-Mercaptopurine (po)  6-Mercaptopurine (po)  Methotrexate (Intrathecal |
| **ALLTogether Pilot Protocol, intermediate risk (low and high) and HR** | | | | | |
| **Treatment phase**  Treatment week | **Induction**  Week 0–4 | **Consolidation I**  Week 5–10 | **Consolidation II**  Week 11–17 | **Delayed intensification**  Week 18–24 (IR high: –29) | **Maintenance I** |
| **Peg-asparaginase treatment^a^**  Doses planned  Treatment week no. | 2 doses  1 + 3 | 3 doses  5 + 7 + 9 | 3 doses (IR-high and HR only)  11+13+ 15 |  |  |
| **Other treatment** | INDUCTION A^b^: Dexamethasone (po)  Vincristine (iv)  Methotrexate^c^ (Intrathecal)  INDUCTION B^d^:  + Daunorubicine (iv) | 6-Mercaptopurine (po)  Cytarabine (iv)  Cyclophosphamide (iv)  Methotrexate (Intrathecal)  Vincristine (iv) | 6-Mercaptopurine (po)  Methotrexate (high dose, iv)  Methotrexate (Intrathecal) | Dexamethasone (po/iv)  Vincristine (iv)  Doxorubicin (iv)  Methotrexate (Intrathecal)  6-Mercaptopurine (po)  Cytarabine (iv) ^e^  + Cyclophosphamide (iv) ^e^ | 6-Mercaptopurine (po)  6-Mercaptopurine (po)  Methotrexate^d^ (Intrathecal |
| Abbreviations: ALL, acute lymphoblastic leukemia; Peg, polyethylene glycol; im, intramuscular; IR, intermediate risk; iv, intravenous; po, per os (by mouth); SR, standard risk  ^a^ Dose: <16 years: 1500 IU/m^2^ (iv), ≥ 16 years: 1000 IU/m^2^ (iv).  ^b^ NCI standard risk (< 10 years, white blood cell count <50 x 10^9^/L, B-cell ALL).  ^c^ +Cytarabine and Prednisolone (intrathecal) for patients with leukemic contamination of the cerebrospinal fluid/CNS-involvement at diagnosis  ^d^ NCI high risk (≥10 years, white blood cell count ≥50 x 10^9^/L, T-cell ALL).  ^e^ Only IR-low | | | | | |

| **Table S3: Risk Stratification as per Treatment Protocol** | |  |
| --- | --- | --- |
| **Risk-group** | **ALLTogether Pilot Protocol** |  |
| **Standard-Risk** | No T-ALL  No HR-genetics (including: KMT2A/MLL gene fusions, near haploidy (<30 chromosomes), low hypodiploidy (30-39 chromosomes), iAMP21, t(17;19)/TCF3-HLF)  MRD not detectable Day 29 (end of induction)  No CNS3 / TLP+  No ABL-class fusion^a^ |  |
| **Intermediate-Risk** | MRD < 5% Day 29 (detectable or not fulfilling criteria for not detectable)    **IR-low**  <16 years at diagnosis  No CNS-involvement (CNS3/TLP+ with increased cell count or clinical)  No HR-genetics (as above)  MRD response:   - ETV/RUNX1 (MRD Day 29 < 0.1%) or - High hyperdiploid (MRD Day 29 < 0.03%) or - B-other + CNA good risk^b^ (MRD Day 29 < 0.05%) - T-cell (MRD not detectable Day 78)     ***IR-high***  All patients not fulfilling SR, IR-low or HR-criteria^c^ |  |
| Abbreviations: ALL, acute lymphoblastic leukemia; BCP, B-cell precursor ALL; CSF, cerebrospinal fluid; HR, high-risk; IR, intermediate risk; MRD, minimal residual disease; SR, standard risk; TLP+, Traumatic lumbar puncture with leukemic blasts; WBC, white blood cell  ^a^ ABL-class fusions = Fusion genes involving ABL1, ABL2, PDGFRB and CSF1R except t(9;22)/BCR-ABL1.  ^b^ Copy-number alteration (CNA) good-risk:   - No deletions affecting BTG1, CDKN2A/B, EBF1, ETV6, IKZF1, PAX5, RB1, and PAR1 - Isolated deletion of BTG1 or ETV6 or PAX5 deletion - Only two deletions - ETV6 and BTG1, ETV6, and CDKN2A/B, ETV6 and a PAX5 deletion   ^c^ High-risk criteria:   - BCP and MRD ≥5% Day 29 - T-cell and MRD ≥5% Day 29 (not patients <16 years with undetectable MRD Day71) - MRD Day 71 ≥0.05% (≥0.01% for NCI HR patients) - Poorly responding extramedullary disease at the end of Consolidation 1 | | |
