# Supplementary material for: Increase in peg-asparaginase clearance as a predictor for inactivation in patients with acute lymphoblastic leukemia
Source: Leukemia. 2024 Jan 29;38(4):712–9. doi: 10.1038/s41375-024-02153-6 (PMC10997509; doi:10.1038/s41375-024-02153-6)
Supplement: Supplementary file 2 — Supplementary 2 [file 41375_2024_2153_MOESM2_ESM.docx]

**Supplementary 2**

**Supplementary Methods: PK model building**

**1. Structural model**

The pharmacokinetics of peg-asparaginase are characterized by an increased clearance within the dosing interval as a consequence of depegylation, in addition to a potential increased clearance over the treatment period in some subjects due to development of inactivation. To capture these patterns, different compartment models for distribution and elimination were assessed based on the asparaginase enzyme activity (AEA) measurements. Models were fitted through the nonlinear mixed effects software NONMEM version 7.4.4 (ICON Development Solutions), using the Laplacian estimation method with interaction. Initially, the time-dependent increase in PEG-asparaginase clearance after first dose was modeled as (I) linear, (II) exponential, (III) Hill or (IV) Weibull functions.

In addition, transit compartment models were tested, where a chain of compartments was representing the process of depegylation in the central compartment, i.e. the amount of AEA in plasma was the sum of the amounts in the transit compartments. Clearance could occur from all transit compartments.^1^ The number of transit compartments was fixed to n=10. The increase in AEA clearance within the dosing interval was explored to be dependent on time after last dose through (I) linear or (II) exponential functions, or as (III) an additional clearance parameter from the last compartment only, as described by Wurthwein et al.^1^ To evaluate the increased clearance over multiple dosing administrations observed in a subset of patients, an increased transport rate through the chain of compartments was evaluated as a function of time from start of treatment through (I) linear, (II) exponential, (III) Hill and (IV) power functions on the transit parameter (Qtr), with and without a time delay.

For intramuscular (IM) administrations, both a first-order and a transit compartment absorption model were evaluated.^2^ To account for samples below the LLOQ (≤5 U/L) or above ULOQ (≥1000 U/L), the M3 method was applied to estimate the maximum likelihood of the AEA being outside the analytical quantification limits.^3^

**2. Stochastic component**

Inter-individual variability (IIV) was evaluated on all structural parameters through a log-normal distribution, with the exception of the delay parameter for which also a uniform distribution was assessed. Potential covariances between clearance, volume of distribution (V), and Qtr were also explored. Residual error models were tested to be additive, proportional and additive plus proportional on both normal and log-transformed data.^4^ In addition, a time-dependent increase in the residual error was assessed.^5^

A mixture model was allowed to estimate two subgroups: one in which Qtr was constant over time and one in which Qtr increased as a function of time. Of note, the model estimation of patient allocation was not informed about the clinical outcome, i.e., hypersensitivity yes/no.

**3. Covariate component**

The impact of covariates on PK parameters was evaluated. Allometric scaling based on body weight was applied, using the fixed exponents 0.75 and 1 for the clearance and V parameters, respectively. The influence of age was additionally assessed on both initial clearance and increase in clearance.

**Supplementary Results: PK model building**

**1. Structural model**


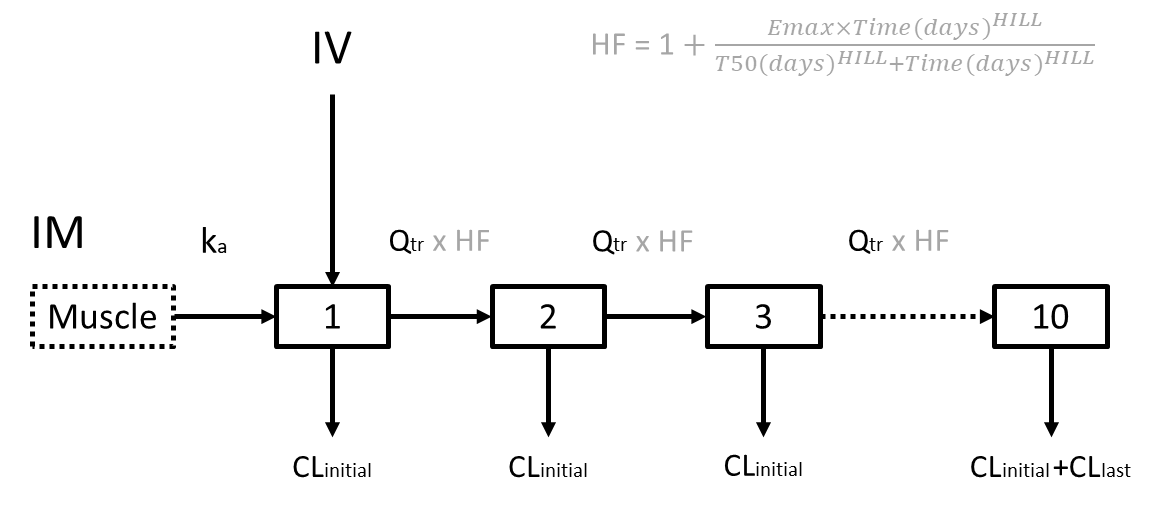


**Figure S1** Schematic overview of the pharmacokinetic model. The previous model published by Würthwein et al. (22) was further extended to describe one group of patients with constant transit clearance (Qtr; HF=1) and one group of patients with increased clearance over dosing occasions (HF time-dependent). The time-dependent HF described an increased transit rate through the chain of compartments. The model-predicted AEA level is the total amount in compartments 1-10 / V. *CL_initial_ initial clearance value, CL_last_ additional clearance value, Qtr intercompartmental transit clearance, HF time-dependent scaling factor for Qtr.*

A transit model with a chain of 10-compartments was used to describe the peg-asparaginase data **(Figure S1**). The model structure included an initial clearance (CL_initial_) out of each compartment in addition to an additional clearance (CL_last_) from the last compartment.

The uptake of peg-asparaginase from the IM administration into the systemic circulation was best described by an absorption model with a first-order rate constant (k_a_). Volume of distribution was estimated at 4.94 L/70 kg (i.e., 2.28 L/25 kg, 3.84 L/50 kg) and CL_initial_ at 0.20 L/day/70 kg (i.e., 0.07 L/day/25 kg, 0.14 L/day/50 kg) (**Table S1**). A mixture model component estimated the probability that patients belonged to one of two subpopulations; i.e. a group with either constant (mix1, constant inter-compartmental Q_tr_ over time), or increased (mix2, increased Q_tr_ over time) Q_tr_, by scaling Q_tr_ through a Hill function dependent on time (Eq. 1). In a posthoc step each patient was assigned to the group with their highest probability.

(Eq. 1) HF_mix1_= 1

HF_mix2_=(1+(E_max_*T^HILL^)/(T50^HILL^ + T^HILL^))

*for k = 1:* DADT(k) = - ka*A(k)

*for k = 2 to n – 1*: DADT(k) = ka *A(k-1) - (CL_initial_/V+Q_tr_*HF/V)*A(k)

*for k = n:* DADT(k) = Q_tr_*HF/V*A(k-1) - (CL_initial_/V+CL_last_/V)*A(k)

Here E_max_ represents the maximum relative increase in Q_tr_, T is time in days since start of treatment, T50 represents the time after treatment at which half of this maximum increase is reached, the Hill coefficient dictates to what degree an increase in time influences the increase in Q_tr_.

**2. Stochastic component**

Addition of a log-normal IIV was found to give a statistically significant improvement for V, CL_initial_, as well as for the Hill coefficient and T50. None of the evaluated covariates and covariances improved model fit. An additive error on log-transformed data was selected to describe residual unexplained variability. Estimation of separate additive errors for the two mixture groups improved parameter precision as well as the visual predictive checks (VPCs) and was therefore included in the final model. All parameters were estimated with good precision, with the exception of CL_last_ for which the relative standard error (RSE) was 60.6% (as calculated by NONMEM as: RSE = (SE/Final parameter estimate)*100).

**Table S1**: Pharmacokinetic parameters

| **Parameter** | **Definition** | **Typical values (%RSE)*** |
| --- | --- | --- |
| V [L] | Volume of distribution | 4.94 (5.4) |
| CL_initial_ [L/day] | Initial clearance | 0.202 (4.3) |
| CL_last_ [L/day] | Additional clearance from last compartment | 198 (60.6) |
| Q_tr_ [L/day] | Transit clearance | 1.34 (3.4) |
| ka [1/day] | Absorption rate constant | 0.233 (9.2) |
| F | Bioavailability for IM administration | 0.518 (4.3) |
| P_mix1,constant_ | Probability to have constant Q_tr_ | 0.755 (4.5) |
| E_max_ | Maximum relative time-dependent increase in Q_tr_ | 58.1 (22.9) |
| T50 [days] | Time to half of E_max_ (maximum increase in Q_tr_) | 49.6 (7.7) |
| HILL | Sigmoidicity factor for time-dependent increase in Q_tr_ | 13.1 (17.3) |
| IIV V [CV%] | CV in volume of distribution | 41.9% (8.1) |
| IIV CL_initial_ [CV%] | CV in clearance initial | 33.6% (7.5) |
| IIV T50 [CV%] | CV in T50 | 72.5% (9.8) |
| IIV HILL [CV%] | CV in HILL | 119.9% (17.2) |
| Additive error mix 1 [IU/L] [σ] | Additive residual unexplained error, mixture 1 (constant Q_tr_) | 0.279 (1.2) |
| Additive error [IU/L] mix 2 [σ] | Additive residual unexplained error, mixture 2 (increased Q_tr_) | 2.06 (0.5) |

* Values scaled to a 70 kg patient. CV = Coefficient of variation expressed as $\sqrt{e^{\omega^{2}}-1}$

**2. Model evaluation**

The mixture model allocated each patient to one of the two subgroups in a posthoc step, according to which mixture population of Q_tr_ (Eq. 1) the patient was most probable to belong to. The patients with high probability to belong to Q_tr,mix1_ were typically those without inactivation and those with high probability to belong to Q_tr,mix2_ were those identified to have inactivation.

Sensitivity of the model classification was calculated as the True Positives / (True Positives + False Negatives). True positives represent the number of patients that had a clinical inactivation and were classified as having increased clearance over the dosing occasions (mix2, increased Q_tr_ over time), whereas false negatives were the patients that had a clinical inactivation but were classified to have stable clearance over the dosing occasions (mix1, stable Q_tr_ over time). Specificity of the model classification was calculated as the Specificity = True Negatives / (True Negatives + False Positives). True negatives represent the number of patients that had no clinical inactivation and were classified as having no increased clearance over the dosing occasions (mix1, stable Q_tr_ over time), whereas false positives were the patients that had no clinical inactivation but were classified to have increased clearance over the dosing occasions (mix2, increased Q_tr_ over time). The sensitivity and specificity of the model to assign an increased clearance to patients with an inactivation response was 93% and 86%, respectively.

The VPCs demonstrate that the model captures the time profile of the continuous data well during the first cycle and the general trend at later time points (**Figure S2**). The mixture group assignment information in the posthoc step was used to separate the observed and simulated data. In other words, the individuals of the original dataset and simulated dataset were separated according to their most probable subpopulation to create the two VPC panels representing stable and increased clearance over time.

The Goodness-of-Fit plots how a balanced distribution of conditional weighted residuals (CWRES) centered around zero over time and with the majority of the values within the 2-standard deviation.

3 patients (~7%) that were clinically classified with an inactivation response, were labeled as having stable clearance in the posthoc step. For two of these patients there were several weeks of measurements missing between the second last AEA and the AEA measurement that confirmed an inactivation response. For one patient the AEA that indicated inactivation was followed by an AEA that indicated no inactivation.

**A**


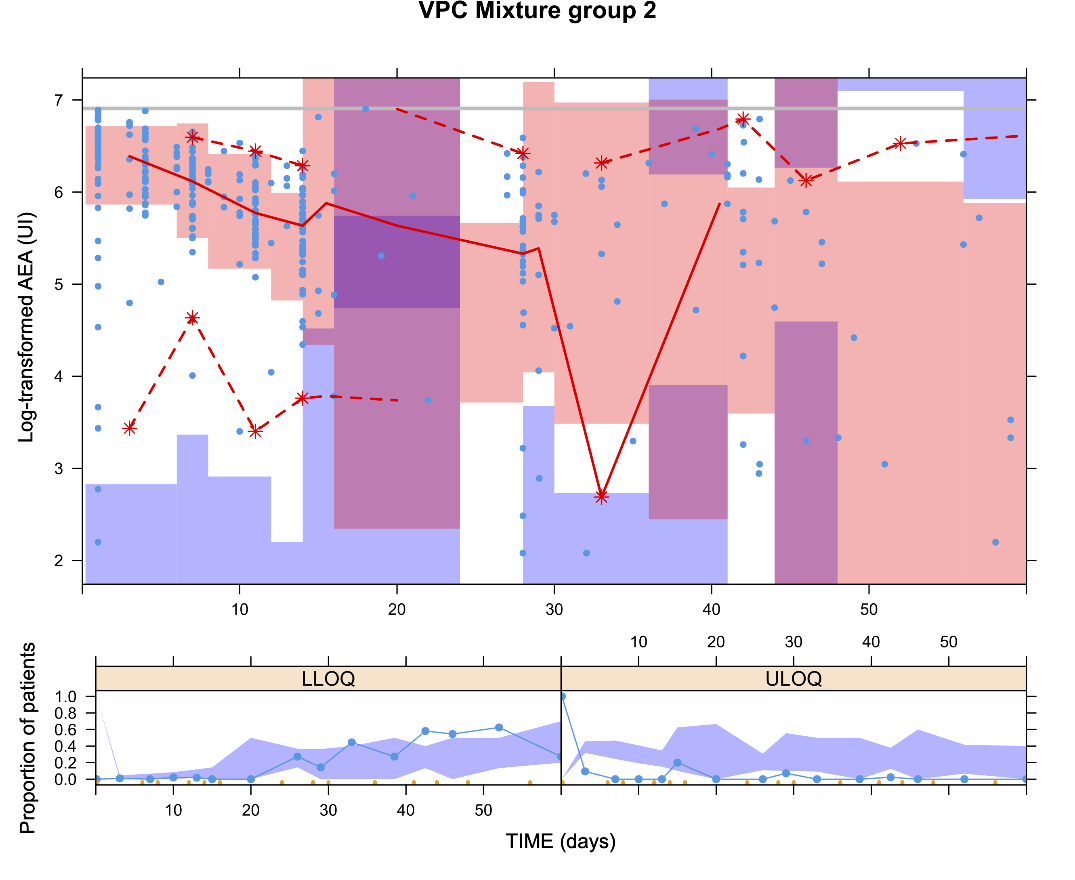


**B**


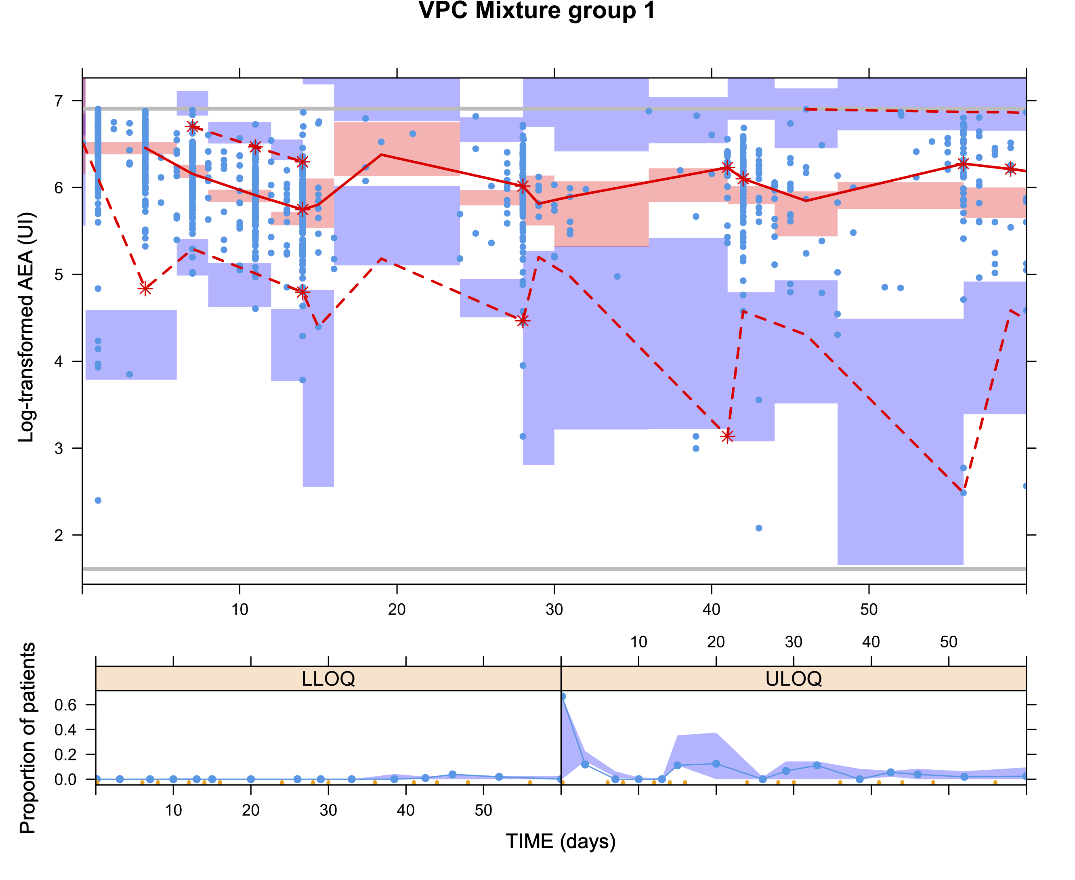


**Figure S2: Visual Predictive Check of pharmacokinetic model.** (A) mixture group 1: constant clearance (B) mixture group 2: increased clearance over time. Time in days. Upper panels: the blue circles (○) represent log-transformed observed AEA (UI), the red solid line (—) represent the median, whereas the red dashed lines (---) represent the 5th and 95th percentiles of the observed data. The shaded areas are the simulated 95% confidence intervals for the median, 5th and 95th percentiles (n = 500). Lower panels: the blue circles (○) represent the proportion of patients outside the limits of quantification and the shaded areas represent the simulated 95% confidence intervals (n = 500).


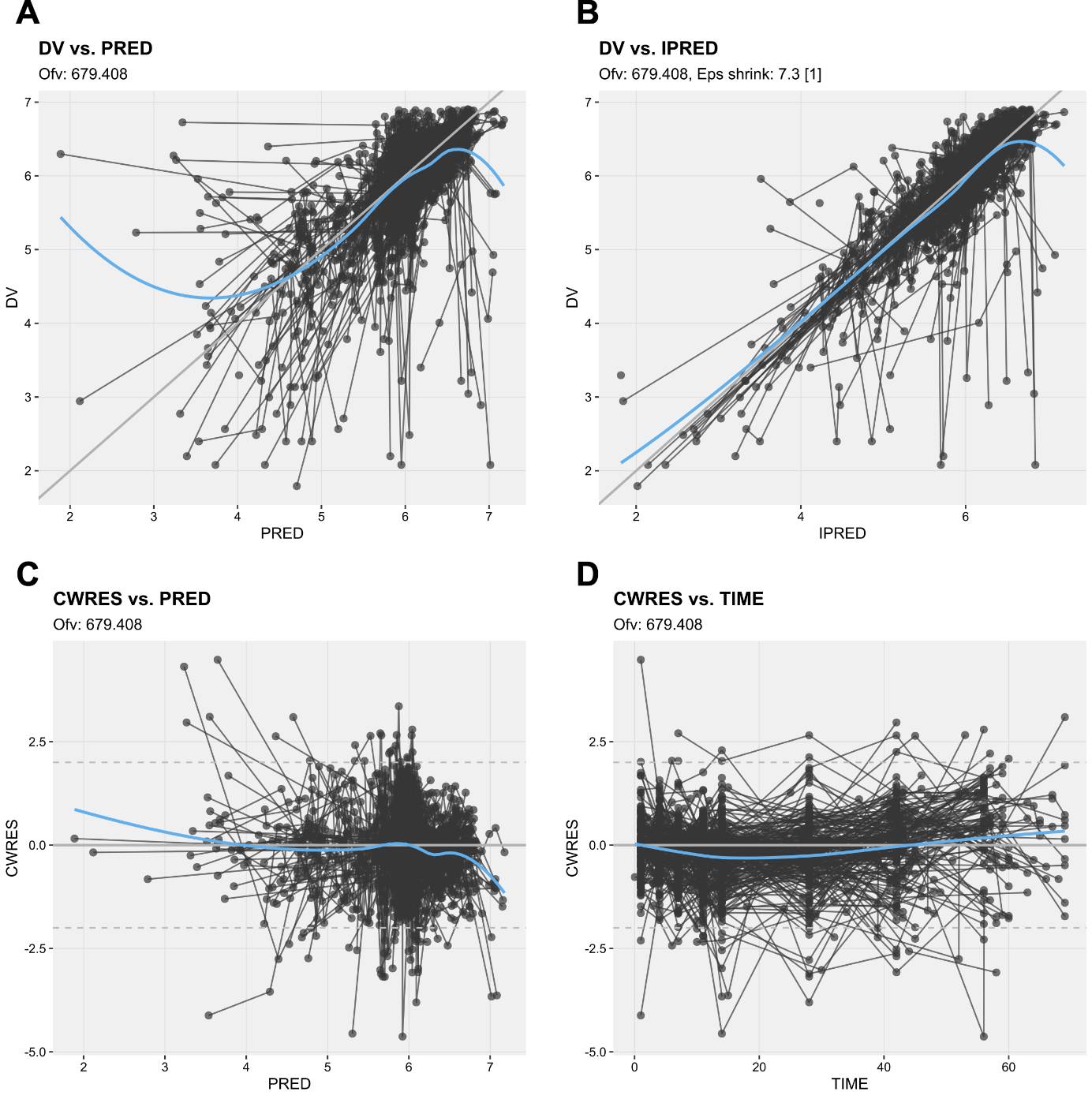


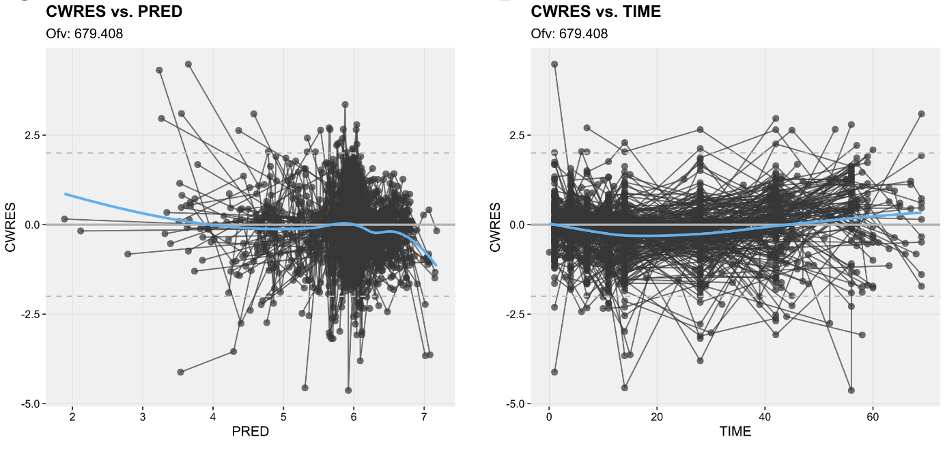


**Figure S3** **Goodness-of-Fit plots for pharmacokinetic model.** A: CWRES versus PRED plot. B: CWRES versus TIME plot. *CWRES = conditional weighted residual error.*


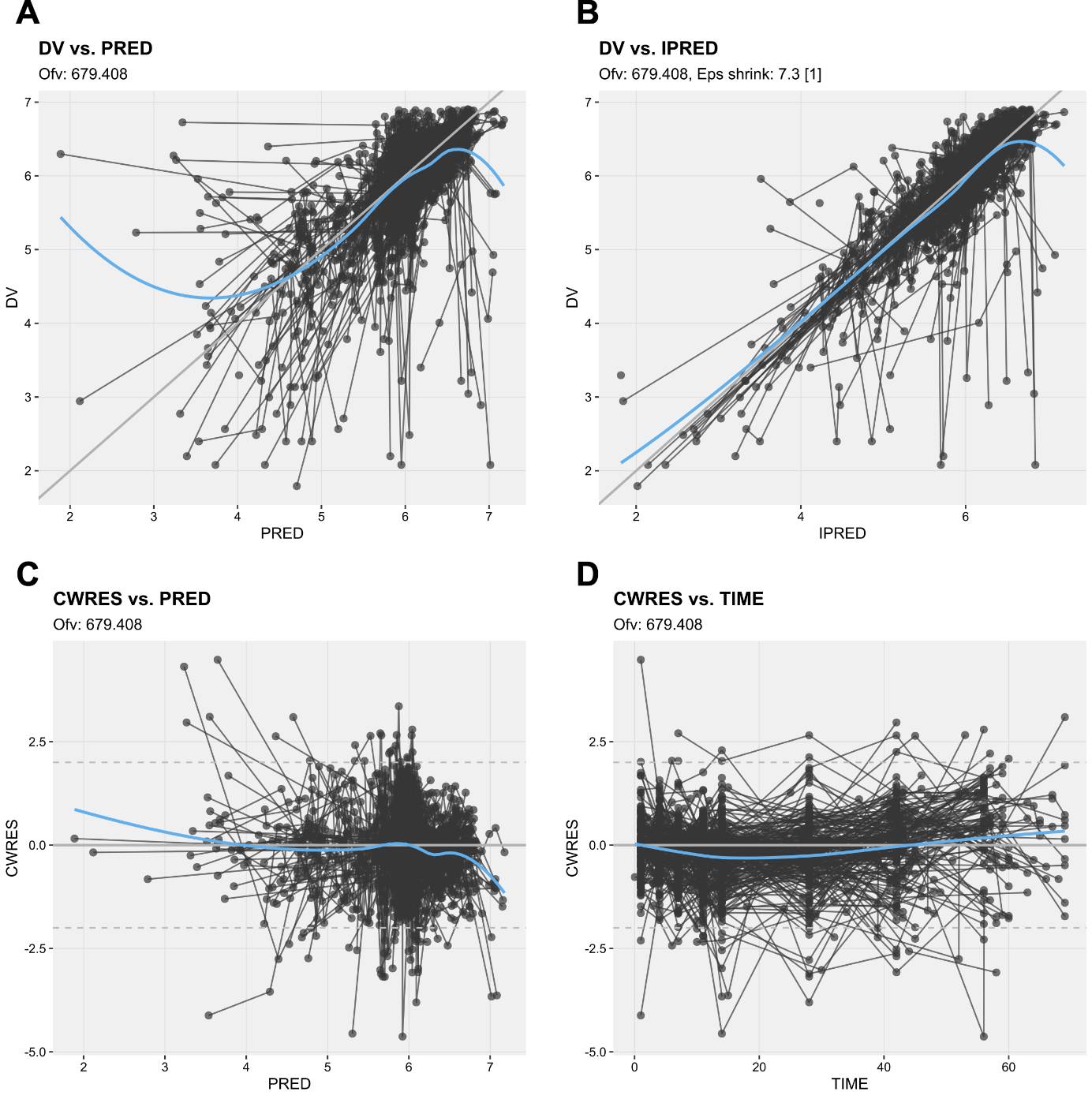


**References**

1. Würthwein G, Lanvers-Kaminsky C, Hempel G, et al. Population Pharmacokinetics to Model the Time-Varying Clearance of the PEGylated Asparaginase Oncaspar(®) in Children with Acute Lymphoblastic Leukemia. *Eur J Drug Metab Pharmacokinet*. Dec 2017;42(6):955-963. doi:10.1007/s13318-017-0410-5

2. Savic RM, Jonker DM, Kerbusch T, Karlsson MO. Implementation of a transit compartment model for describing drug absorption in pharmacokinetic studies. *J Pharmacokinet Pharmacodyn*. Oct 2007;34(5):711-26. doi:10.1007/s10928-007-9066-0

3. Bergstrand M, Karlsson MO. Handling data below the limit of quantification in mixed effect models. *Aaps j*. Jun 2009;11(2):371-80. doi:10.1208/s12248-009-9112-5

4. Proost JH. Combined proportional and additive residual error models in population pharmacokinetic modelling. *Eur J Pharm Sci*. Nov 15 2017;109s:S78-s82. doi:10.1016/j.ejps.2017.05.021

5. Silber HE, Kjellsson MC, Karlsson MO. The impact of misspecification of residual error or correlation structure on the type I error rate for covariate inclusion. *J Pharmacokinet Pharmacodyn*. Feb 2009;36(1):81-99. doi:10.1007/s10928-009-9112-1
